# Mitochondrial Variants in Schizophrenia, Bipolar Disorder, and Major Depressive Disorder

**DOI:** 10.1371/journal.pone.0004913

**Published:** 2009-03-17

**Authors:** Brandi Rollins, Maureen V. Martin, P. Adolfo Sequeira, Emily A. Moon, Ling Z. Morgan, Stanley J. Watson, Alan Schatzberg, Huda Akil, Richard M. Myers, Edward G. Jones, Douglas C. Wallace, William E. Bunney, Marquis P. Vawter

**Affiliations:** 1 Department of Psychiatry & Human Behavior, University of California Irvine, Irvine, California, United States of America; 2 Molecular and Behavioral Neuroscience Institute, University of Michigan, Ann Arbor, Michigan, United States of America; 3 Department of Psychiatry, Stanford University, Palo Alto, California, United States of America; 4 Hudson Alpha Institute for Biotechnology, Huntsville, Alabama, United States of America; 5 Neuroscience Center, University of California Davis, Davis, California, United States of America; 6 Molecular and Mitochondrial Medicine and Genetics, University of California Irvine, Irvine, California, United States of America; Freie Universitaet Berlin, Germany

## Abstract

**Background:**

Mitochondria provide most of the energy for brain cells by the process of oxidative phosphorylation. Mitochondrial abnormalities and deficiencies in oxidative phosphorylation have been reported in individuals with schizophrenia (SZ), bipolar disorder (BD), and major depressive disorder (MDD) in transcriptomic, proteomic, and metabolomic studies. Several mutations in mitochondrial DNA (mtDNA) sequence have been reported in SZ and BD patients.

**Methodology/Principal Findings:**

Dorsolateral prefrontal cortex (DLPFC) from a cohort of 77 SZ, BD, and MDD subjects and age-matched controls (C) was studied for mtDNA sequence variations and heteroplasmy levels using Affymetrix mtDNA resequencing arrays. Heteroplasmy levels by microarray were compared to levels obtained with SNaPshot and allele specific real-time PCR. This study examined the association between brain pH and mtDNA alleles. The microarray resequencing of mtDNA was 100% concordant with conventional sequencing results for 103 mtDNA variants. The rate of synonymous base pair substitutions in the coding regions of the mtDNA genome was 22% higher (p = 0.0017) in DLPFC of individuals with SZ compared to controls. The association of brain pH and super haplogroup (U, K, UK) was significant (p = 0.004) and independent of postmortem interval time.

**Conclusions:**

Focusing on haplogroup and individual susceptibility factors in psychiatric disorders by considering mtDNA variants may lead to innovative treatments to improve mitochondrial health and brain function.

## Introduction

Mitochondrial DNA (mtDNA) mutations can alter functions involving apoptosis, calcium buffering and signaling, and the brain's capacity to meet critical energy oxidative phosphorylation (OXPHOS) demands. The inheritance of mtDNA is normally matrilineal [1]. High rates of maternal offspring of SZ [2], [3], [4], [5] and BD [6] compared to paternal rates have been primarily found in family studies [7], [8] but not in the general population [9], [10], [11]. This supports the hypothesis that increased risk for these disorders might be related to mitochondrial dysfunction. Failure to find a pattern of maternal inheritance as well as evidence of paternal inheritance have also been reported in SZ and BD [12], [13].

Tissues with high energy requirements seem to accumulate mtDNA mutations more readily; thus, in general, the accumulation of mtDNA mutations is proportional to their metabolic rate [14]. Multiple mechanisms across the life span exist for transmission of mtDNA mutations [15], [16]. A cell may become heteroplasmic (increase of intra individual sequence variability in mtDNA) through a germ line or an acquired somatic mutation; thus, heteroplasmy may be inherited or acquired [17], [18]. MtDNA has been suggested as a potential “weak point” of the genome [19] due to susceptibility to somatic deletions as mtDNA is not protected by histones and lacks the repair machinery associated with nuclear DNA [20] which is one reason there is a higher rate of mutation or base pair substitutions in mtDNA compared to nuclear DNA. Heteroplasmic events in brain appear to be common in several studies [21], [22], [23], [24], [25]. Small changes in the level of heteroplasmy may be clinically relevant, and can appear in vulnerable cells and tissues in neurodegenerative disorders such as Parkinson's, Huntington's, and Alzheimer's Disease [16], [26], [27], suggesting a critical threshold of heteroplasmic mutation above which functional deficits and neuropathological damage occurs.

### Evidence for dysfunction of mitochondria in psychiatric disorders

Psychiatric symptoms have been documented in subjects with mitochondrial disease [28], [29], [30]. Conversely, increased psychiatric symptoms have been associated with declines in mitochondrial functional activity [31], [32], [33], [34]. The neuroimaging literature suggests that energy metabolism abnormalities are widespread in the brains of subjects with SZ [35], [36], [37], [38], BD [39], [40], [41], [42], and MDD [43].

### Common, novel, and ethnic specific defining haplogroup mitochondrial SNPs are associated with psychiatric disorders

Sequence variations in the mitochondrial genome are implicated in the pathogenesis of SZ and BD [44], [45], [46], [47], [48], [49], [50], [51], [52], [53], [54], [55], [56], [57], [58]. ND1, ND3, ND4, and ND5 SNPs have been associated with SZ or BD [51], [52], [59], [60]. An interesting SNP, the ND3 A10398G polymorphism, may be related to lithium response in BD [61] as well as increased pH in cybrid cells [62].

Ethnic specific defining haplogroup SNPs are also reported to be associated with psychiatric disorders. The sub-haplogroup F1ba of the haplogroup N, may harbor a haplotype risk factor for atypical psychosis [63] and in an Israeli sample of SZ there was an over-representation of the HV mtDNA haplogroup [64]. Several attempts have been made to assess association of certain mtDNA genetic variants with BD [44] in haplogroup T. A recent report indicated that the number of synonymous substitutions was increased in mtDNA obtained from blood samples in SZ [58], independent of haplogroup.

Based upon the studies implicating mitochondrial dysfunction in psychiatric disorders, the present study measured mtDNA variants in brain tissue, including substitutions, synonymous and non-synonymous, and rare variants, which could predispose to BD, MDD, and SZ. Secondly, we compared heteroplasmy detection by microarray using a published method [65] to conventional methods. Thirdly, this study examined the association between brain pH and mtDNA alleles. The hypothesis that mtDNA variants might influence brain pH is supported by the haplogroup defining ND3 A10398G polymorphism associated with an increased pH in cybrid cells [62]. Further, microarray studies have demonstrated differential nuclear-encoded mitochondrial gene expression [45], [66], [67] as well as mitochondrial gene expression [67] related to mean group pH differences. ^31^P magnetic resonance spectroscopy findings have also indicated a decrease in intracellular pH in BD [68] increasing the potential relevance of such an effect on psychiatric disorders.

## Results

### Microarray resequencing arrays and capillary electrophoresis sequencing of mtDNA

The Affymetrix Genetic Sequence Analysis Software (GSEQ) assigned high quality base pair sequencing calls (T,C,G,A) to 98.2% of the mtDNA genome on all 77 chips analyzed. There was a 100% concordance between microarrays resequencing the same individual repeated de novo. The call rate did not depend on the source of starting genomic DNA, as call rates from 8 freshly prepared lymphocyte samples were also 98.2% on average in the investigator's laboratory. Three individual mtDNA genomes were previously sequenced on an ABI 3130 capillary electrophoresis (CE) sequencer. All 103 calls that were different from the rCRS were 100% concordant between microarray and CE sequencing in these three individuals. The present mitochondrial microarray resequencing results are consistent with additional experimental verification from other investigators showing at least 99.99% microarray accuracy compared to sequencing [65], [69], [70], [71], [72], [73], [74], [75]. In addition, the microarray procedure detected the known heteroplasmic mutation A5793G with 45% heteroplasmy [65]. The same subject was found to be 30% heteroplasmic by PCR-RFLP analysis. Taken together, these initial results confirmed the mitochondria resequencing array is a useful tool for resequencing.

### Haplogroup differences in psychiatric disorders

**Figure 1:**
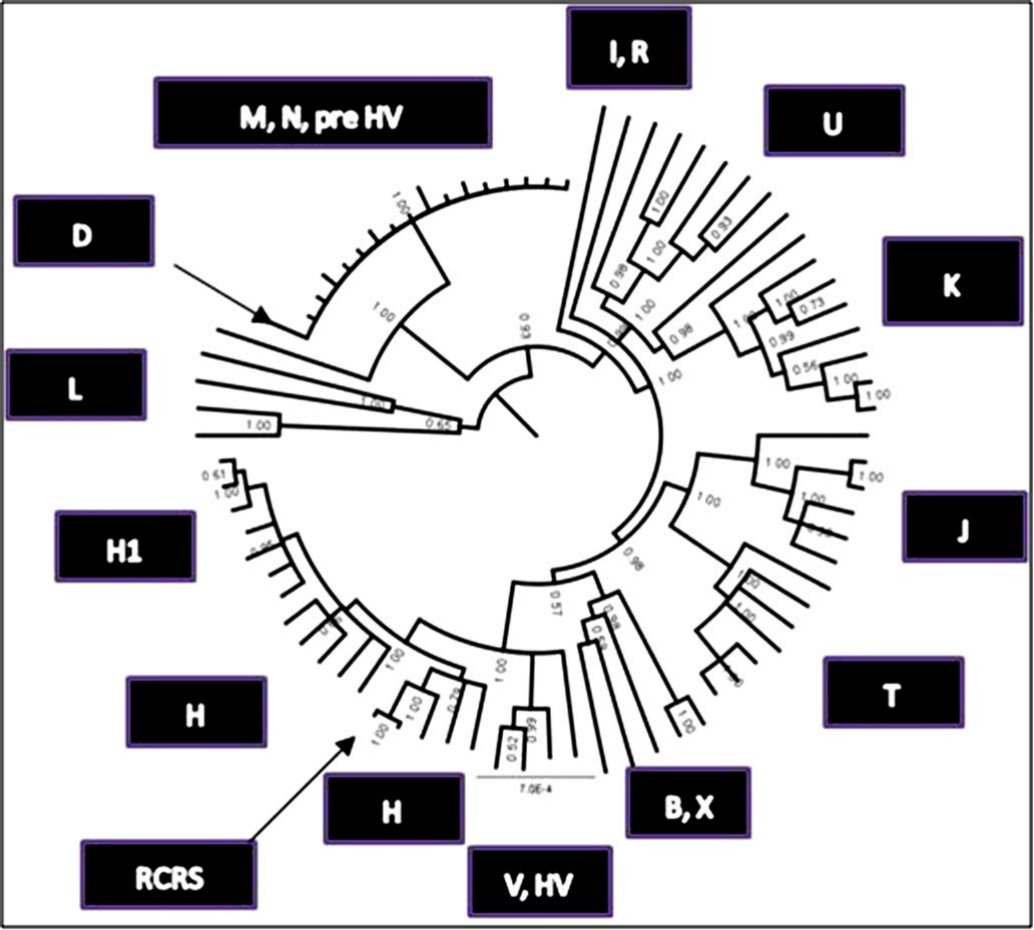
Phylogenetic tree of human mtDNA coding sequence variants demonstrating regional association of haplogroups using mtDNA sequence from DLPFC. Haplogroups, groups of related haplotypes, are derived from a founding haplotype, harboring characteristic mtDNA sequence polymorphisms. Each haplogroup is designated by a letter in this tree, the RCRS is the reference sequence; the ticks around the perimeter of the circle represent the individual mtDNA sequences from DLPFC. The faint numbers represent the probability of support for the branches which was 0.9–1.The mtDNA haplogroups have proven to be highly geographically associated [109]. The haplogroup assignments were calculated at MITOMASTER (http://mammag.web.uci.edu/twiki/bin/view/Mitomaster/WebHome) using all mtDNA sequence base pairs.

Upon completion of the resequencing arrays, the 77 mtDNA sequences from DLPFC were aligned with the rCRS. A phylogenetic tree (Fig. 1) was reconstructed with the MRBAYES program, which is a variant of the Markov chain Monte Carlo method [76], [77].

The rCRS clustered far from the ancestral L haplogroup, and was correctly placed within the H haplogroup with an H2 subject as the nearest neighbor (Fig. 1). Individuals within each haplogroup are joined neighbors except for the node labeled M, N1B1, pre HV, which placed these three haplogroups as closest neighbors. One reason these haplogroups did not cluster separately as expected was that each of these samples also carried a high rate of synonymous substitutions (shown in the next section). The majority of 17 individuals (88%–100%) in this cluster carried mtSNPs different from rCRS at the following positions: 9540, 10652, 12705, 14783, 15043, 16311, 16325. The underlined mtSNPs were found in the M, N, or D haplogroups (/http://www.mitomap.org/mitomap-phylogeny.pdf). The 10652 mtSNP was found in one L2b individual (Mitokor 568 sequence [78]); 16325 and 16311 were not found in a specific haplogroup.

Previous haplogroup findings that were reported for BD [44] indicated a slight over-representation in the T haplogroup, thus the T haplogroup was explored further in the current dataset. The opposite finding emerged, controls were significantly over-represented in haplogroup T compared to BP+SZ (p = 0.04 permuted p-value). There was complete concordance of findings for all nine SNPs defining this haplogroup (709, 1888, 4917, 8697, 10463, 13368, 14905, 15607, 15928). This **c**oncordance illustrates confidence for haplogroup assignment using microarray resequencing.

Another exploratory difference in the distribution of psychiatric groups by haplogroup (Table 1) was seen in analysis of the combined BD+SZ distribution of subjects found more frequently in the pre HV group (Table 2) compared to all haplogroups (Chi square = 13.7, df (6), p = 0.032). To further test for association of mtDNA SNPs we analyzed the SZ, BD, MDD mtDNA SNPs dataset (484 polymorphisms) using PLINK [79].

**Table 1:** The haplogroup determination of mtDNA derived from DLPFC showed 31 diverse haplogroups that could be reconstructed.

Haplogroup	BD	Control	MDD	SZ
B2	1	0	0	0
B4a	0	1	0	0
B4b	0	1	0	0
C2	0	1	0	0
D1	0	0	1	0
H1	1	5	0	1
H2	0	0	0	3
H3	0	3	2	1
HV	0	2	0	0
I1	0	1	0	0
J1c	1	2	0	1
J2	1	0	0	0
J3	0	1	0	0
K1	2	2	1	2
K2	0	1	0	0
L1b	0	0	0	1
L1c	0	1	0	0
L2a	0	2	0	0
M	0	2	1	2
N1B1	1	1	3	1
Pre HV	3	1	0	1
R1	0	1	0	0
T1	0	1	0	0
T2	0	3	2	0
T2a	0	1	0	0
U2	0	0	1	0
U5a	1	2	2	0
U6	0	0	1	0
V2	0	0	0	1
V3	0	1	0	0
X2	1	0	1	0
Total	12	36	15	14

**Table 2:** The haplogroup determination of mtDNA derived from DLPFC was condensed from Table 1 and showed that BD and SZ were over-represented in Pre HV haplogroup and MDD was over-represented in the “other” haplogroups.

Haplogroup×Psychiatric Diagnosis			
Diagnosis	H,HV, V	Pre HV	Others
**Bipolar Disorder**	1	3	8
**Control**	11	1	24
**Major Depressive Disorder**	2	0	13
**Schizophrenia**	6	1	7

The chi-square for this table is (chi-square = 13.7 (6), p = 0.032).

### Case-control differences in polymorphisms

An allelic association analysis was conducted across polymorphic sites in mtDNA sequence from the 77 subjects. Permutation analysis (n = 50,000) for each polymorphism was run using PLINK [79] to establish an empirical p-value for SZ v C, MDD v C, and BD v C comparisons. There were 484 nominal polymorphic sites, and in this first pass analysis, all SNPs were calculated for association, without regard to haplogroup specificity or minor allele frequency. Only empirical p-values<0.05 are reported for case-control comparisons (Table 3). There were two 12S rRNA rare polymorphisms found in SZ and not in C. The 12S mtDNA G750A and G1438A SNPs had frequencies of 0.143 and 0.214 in SZ, respectively. Both substitutions have been reported in 2,704 mtDNA sequences at a rate of 0.008 for G750A and 0.032 for G1438A [80]. The empirical p-values for association with G750A and G1438A were 0.037 and 0.012, respectively. The ND2 gene G4769A synonymous substitution showed a significant allelic association with SZ (empirical p value 0.037).

**Table 3:** Mitochondrial polymorphisms were analyzed in PLINK for allelic association with 50,000 case-control permutations to obtain empirical p-values.

Disorder	Position mtDNA (RCRS).	Gene	Allele	Allele Frequency Affected	Allele Frequency Unaffected	Allele 2	p-value permutation) ^a^	World Wide MAF ^b^	Psychiatric MAF/World Wide MAF ^c^	Base	A	G	C	T
BD	114	D-loop	T	0.250	0.030	C	**0.032**	0.0048	51.56	C			1856	9
BD	195	D-loop	C	0.571	0.125	T	**0.007**	0.1779	3.21	T	11		280	1574
BD	10652	ND4L	C	0.333	0.086	T	**0.037**	0.0000		T				2704
BD	16300	D-loop	G	0.167	0.000	A	**0.014**	0.0032	51.70	A	1861	6		
SZ	750	12S rRNA	A	0.143	0.000	G	**0.037**	0.0082	17.42	G	22	2682		
SZ	1438	12S rRNA	A	0.214	0.000	G	**0.012**	0.0321	6.68	A	84	2620		
SZ	4769	ND2	A	0.143	0.000	G	**0.037**	0.0112	12.74	A	30	2674		
MDD	10652	ND4L	C	0.333	0.086	T	**0.026**	0.0000		T				2704
MDD	14668	ND6	T	0.133	0.000	C	**0.042**	0.1118	1.19	C			2432	272
MDD	15043	Cytb	A	0.400	0.143	G	**0.042**	0.4032	0.99	G	777	1927		

The number of individuals with mtDNA alleles shown in the far right columns is from the Ingman mt database (URL http://www.genpat.uu.se/mtDB/). A preliminary odds-ratio was calculated by comparing the affected allele frequency to the Ingman database [80]. All case-control differences were either in the control region, or a synonymous substitution in the third position in coding regions.

For BD, there were three allelic associations significant in the D-loop control region: T114C (p = 0.032), C195T (p = 0.008), and G16300A (p = 0.012). The T114C and G16300A minor alleles are rare at 0.004 and 0.003 in a database of 2,704 mtDNA genomes [80]. Three associations for MDD are shown in Table 3.

### Microarray Heteroplasmy

**Figure 2:**
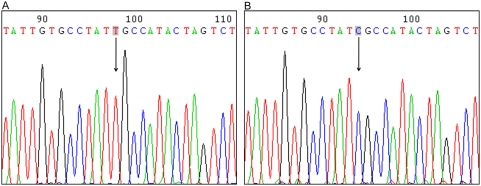
Electropherogram results for direct sequencing of the ND4L T10652C variant. (A) is the ‘T’ allele sequencing result from the lowest heteroplasmic individual used to make the ‘T’ allele clone standard for SNaPshot and allele specific PCR. (B) is the ‘C’ allele sequencing result from the highest heteroplasmic individual detected with microarray (MDD subject from haplogroup D1) used to make the ‘C’ allele clone standard.

The present microarray data shows the ND4L T10652C variant appears within the N (N1B1, pre HV) and M (D1) haplogroups. There were 17 subjects within these haplogroups that had a heteroplasmy level greater than 50% by microarray calculation as previously described [65]. This ND4L T10652C transition is rare and was not present in a human mtDNA database of 2,704 sequences distributed among major haplogroups [80]. In addition it was not observed in 12 mtDNA samples from control blood measured by microarray resequencing, reducing the likelihood of a microarray artifact. There was a common haplotype for the putative T10652C individuals at four mtSNPs – T10652C, T14783C, G15043A, and T9540C for 15 of 16 subjects. These three mtSNPs appear early in the phylogenetic mtDNA evolutionary tree at L2b, and the roots of M, and N. A post hoc analysis of each SNP was run separately and pooled for the three psychiatric groups (SZ, BD and MDD) and compared to controls. A trend for an association for each SNP was found (T14783C p = 0.06, G15043A p = 0.07, T9540C p = 0.16). These observations prompted further investigation of the variant by sequencing, SNaPshot, and allele specific PCR. The ND4L T10652C variant was confirmed by direct sequencing of mtDNA for the highest heteroplasmic individual (81% C heteroplasmy by microarray calculation) within the D1 haplogroup who was an MDD subject and had the C allele (Fig. 2). The other 16 individuals within the N (N1B1, pre HV) and M (D1) haplogroups showed the T allele with direct sequencing. The heteroplasmy detection limit of conventional direct CE sequencing is 20%–30% [81], [82], indicating that the microarray results were inflated for these subjects. All subjects were resequenced 100 bp on either side of the ND4L T10652C for insertions or deletions. There were no insertions or deletions found suggesting that the microarray results were not due to probe mismatches. Primers used for sequencing and allelic specific PCR did not amplify genomic DNA extracted from ρ° cells depleted of mtDNA [83].

SNaPshot multiplex was tried for validation of the microarray calls at T10652C. Twenty four subjects with varying heteroplasmy levels by microarray were run and all individuals showed the T allele indicating their heteroplasmy levels, if present, were below the detection limit of this method. Although we ran a standard curve consisting of dilutions from 100% down to 0.1% for the C allele, we were only reliably able to detect 5% or greater heteroplasmy using SNaPshot.

**Figure 3:**
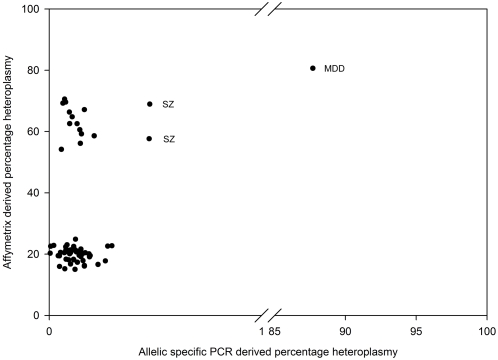
Scatter plot of the heteroplasmy levels of ND4L T10652C calculated from the Affymetrix mitochondrial resequencing chip by method of Coon *et al.* [65] on the y-axis and allelic specific PCR (x-axis truncated).

A more sensitive method for validation of the calculated heteroplasmy from the microarray resequencing data was performed with an allelic specific real-time detection and quantification experiment for the T10652C variant (Fig. 3). The subject with the highest heteroplasmy in the initial microarray experiment (81%) showed similar levels in the confirmation experiment (88%). Because the allelic specific experiment can detect low levels of heteroplasmy (0.25%), we were able to detect several other subjects with heteroplasmy >0.25%. Two SZ subjects (from haplogroups pre HV and M) with 69% and 58% heteroplasmy in the microarray experiment had 0.47% heteroplasmy in the real-time allelic specific qPCR experiment. Despite the failed confirmation of some of the microarray results with 50% and higher heteroplasmy, the three subjects with somewhat higher levels of heteroplasmy were all psychiatric subjects.

### Substitution bias in subjects with psychiatric disorders and controls

One measure of neutrality for mtDNA variants was calculated by comparing the number of non synonymous substitutions (dN) to the number of synonymous substitutions (dS) in each group. Although it is generally accepted that S changes are greater than N changes in mtDNA, we tested for differences between groups in this rate using pair wise comparisons. Values of p<0.05 were considered significant and tabulated (Table 4, column [A] shows the number of significant pair wise comparisons). Table 4 shows the number of times the null hypothesis of strict-neutrality (dN = dS) was accepted in [B] or rejected in favor of the alternative hypothesis (dN<dS, column [A]) using pair wise comparisons for all possible subject combinations of BD-C, SZ-C, MDD-C, and C-C. Occurrences that were significant are counted in column [A]. The higher rate of synonymous substitutions in schizophrenia by 22% compared to the controls was significant (p = 0.0017).

**Table 4:** Codon-based test of purifying selection for pair wise analysis between sequences.

Group	*[A]* dS>dN	*[B]* dN = dS	*[A]/[A+B]*	*[A]/[A+B]/*Control	Chi-Square	p-value
BD	193	239	0.44	(1.11)	2.42	0.119
MDD	202	296	0.40	(1.01)	0.03	0.85
**SZ**	**247**	**257**	**0.49**	**(1.22)**	**9.82**	**0.0017**
Control	297	445	0.40	(1.00)	NA	NA

Values of p<0.05 were considered significant and tabulated; column [A] shows the number of significant pair wise comparisons. Subjects with SZ showed a 22% increase in the number of synonymous substitutions relative to non synonymous mtDNA substitutions in DLPFC (p = 0.0017).

Column [A] is the number of SNPs by group for purifying selection; column [B] is the number of SNPs by group for neutral selection. The column showing the ratio of [A]/[A+B] is to normalize the purifying SNPs by the total SNPs for the group. The chi-square values are calculated for columns [A] and [B] and for SZ group compared to the control group, there was a higher rate of synonymous substitutions.

A second analysis of the overall transition/transversion bias (R) for all DLPFC mtDNA sequences was conducted using the Maximum Composite Likelihood method in MEGA4 [84], [85] for each of the four groups separately. Codon positions included were 1st, 2nd, 3rd. The observed transition/transversion ratios were 2.25 fold higher in SZ compared to C (Table 5). This suggests a higher number of substitutions in DLPFC in SZ compared to controls in the 13 mtDNA transcripts. Two caveats apply to this difference in bias: the overall sample size is small (n = 77) and the total number of transversions in the entire sample was modest (n = 22) which can inflate R. Further, we used a homogeneous model to assume that all sites are variable, and may over estimate the number of substitutions based upon pairs of sequences. With these caveats, Tables 4 and 5 consistently showed the number of substitutions in SZ pair wise comparisons exceeded the rate in control comparisons and the overall R rate is higher in SZ than in C. The present R ratio (transition/transversion) data was examined in the coding region functional grouping level by complex, the rate for R in mitochondrial complex I was 10 fold higher in SZ compared to controls.

**Table 5:** Transition/transversion bias calculation for mtDNA coding sequences shows difference between SZ and control group substitution.

Group	Transition/Transversion Bias (R)	(R)/Control)
**SZ**	**36.61**	**2.25**
BD	13.28	0.81
MDD	21.02	1.29
C	16.27	1.00

* *R* = [A*G*k_1_+T*C*k_2_]/[(A+G)*(T+C)]. Codon positions included were 1st+2nd+3rd. All calculations were conducted in MEGA4 [84].

This result is consistent with the increased synonymous substitution rate in SZ shown in Table 4.

### Association with postmortem pH

Postmortem pH was next analyzed for association with mtDNA SNPs. Association and permutation analysis were conducted in PLINK and the permuted p-values that were most significant for the association of pH were three mtDNA SNPs in tRNA Leu, ND4, and ND5 (association p-values 0.002–0.0004, Table 6). These same three mtDNA SNPs are ethnic-specific haplotype-defining polymorphisms for the super haplogroup cluster (U, K, UK) and all subjects with these three mtDNA SNPs were assigned to these haplogroups in this study using MITOMASTER (www.mitomaster.org). This super haplogroup (U, K, UK) had a significantly higher postmortem pH (7.00±0.18 SD) compared to the remaining subjects pH (6.8±0.18 SD) and the permuted p-value for the pH difference was 0.01 for 5,000 random group assignments for membership in the super haplogroup cluster (U, K, UK).

**Table 6:** pH in postmortem brain showed significant association with three mtDNA SNPs.

Position mtDNA (RCRS)	Base	A*	G*	A#	G#	Location	Amino Change	p-value of pH association empirical
12308	A	2357	347	56	15	tRNA Leu		0.000435
11467	A	2357	347	55	15	ND4	Leu ->Leu	0.000589
12372	G	390	2314	15	58	ND5	Leu ->Leu	0.001118

The empirical p-values were from PLINK with permutation. The three mtDNA SNPs define the super- haplogroup U, K, UK matrilineages. This super-haplogroup demonstrated significantly increased postmortem pH (mean 7.0±0.18 SD) compared to the other haplogroups combined (mean 6.8±0.18 SD); permuted p-value was 0.01 for 5,000 random group tests. This robust association was not altered by differences in PMI, agonal factors, or case-control assignment differences between haplogroups. The PMI was not different between the U, K, UK super haplogroup and the remaining haplogroups, and including PMI in ANCOVA did not reduce the significance of the pH differences (p = 0.0007 with PMI as a covariate).

* The A and G columns refer to Ingman database frequencies (URL http://www.genpat.uu.se/mtDB/), while the A# and G# columns refer to observed frequencies in this study.

**Figure 4:**
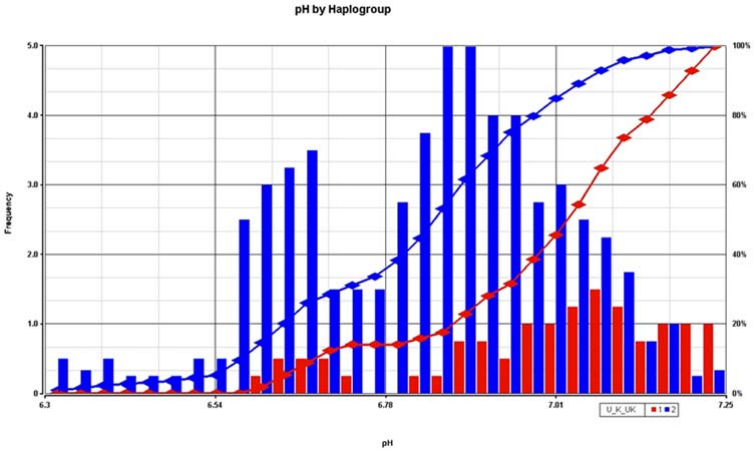
The super haplogroup (U, K, UK) showed a shift in postmortem brain pH, and this finding was significant following permutation analysis. The histogram shows the frequency of the pH (bars) for the super haplogroup (red) compared to all other matrilineages (blue). The accumulated percentage (right y-axis) is shown as two lines with the super haplogroup (red line) and all other matrilineages (blue line). There were no subjects with a prolonged death, or agonal factors as rated according to the Hardy *et al.* scale [98]. PMI differences did not account for this significant effect, as PMI was equivalent between the super haplogroup and other haplogroups.

The present study design intentionally avoided some peri-mortem artifacts by pre-selecting subjects with rapid deaths and without recorded agonal factors in the autopsy database. The pH association did not appear spurious due to differences in assignment of cases and controls as the distribution was equally distributed for psychiatric cases and controls between U, K, UK super haplogroup and the remaining haplogroups. The pH shift towards the right for the U, K, UK super haplogroup and all other subjects (Fig. 4) showed that the lowest nine brain pH readings were all from subjects in the other super haplogroups besides U, K, or UK. Removing the lowest five pH subjects did not change the association of pH nor reduce the bootstrap significance of the difference between haplogroups. However, to exclude if the effect could be primarily related to factors like postmortem interval or agonal factors, we controlled for both effects. The PMI was not different between the U, K, UK super haplogroup and the remaining haplogroups, and including PMI in ANCOVA did not reduce the significance of the pH differences (p = 0.0007 with PMI as a covariate).

## Discussion

The present study analyzed mtDNA sequence data collected from 17 major yet diverse haplogroups residing in Southern California. Over 1.6×10^6^ bp of high quality mtDNA genetic data was generated. Mitochondrial array resequencing data was 99.999% identical to conventional CE sequencing [69] indicating that the mitochondrial resequencing array is a reliable method. The mitochondrial microarray, however, showed a large N call rate, which may be improved by using similar PCR conditions as the National Institute of Standards and Technology. Additionally, changing the base pair calling algorithm [69] could increase the average call rate to >99% across the entire mtDNA genome [69], [70], [86]. In addition, the mitochondrial microarray showed a high heteroplasmy level using the calculation method of Coon *et al.* [65] for the ND4L T10652C SNP which could not be validated entirely with SNaPshot or allele specific PCR.

The main finding relevant to schizophrenia was an increased number of synonymous base substitutions in mtDNA in DLPFC. The first method used a codon purifying selection method [84] in coding regions only, and examined pair wise concordances of sequences to establish, for each group, whether there were synonymous substitutions in codons. The rate of synonymous substitutions in schizophrenia was 22% higher compared to the control rate (p = 0.0017).

The second calculation method was a transversion/transition rate bias (R) [84] performed for each group separately. R was increased 2.25 fold in SZ compared to controls. The present R ratio (transition/transversion) data was examined on individual mitochondrial complex functional grouping levels, the rate for R in mitochondrial complex I was 10 fold higher in SZ compared to controls. This suggests an increase in the number of substitutions in DLPFC in SZ compared to controls preferentially in mtDNA transcripts in complex I. There is a previous report of age related increases of synonymous substitutions in brain [87]. It has been previously demonstrated that there is an increase in the rate of substitutions in somatic mtDNA in brain[21]. Increased synonymous substitutions have also been reported in blood mtDNA in SZ [58]. A possible mechanism could be a result of nuclear gene defects in genes such as POLG. POLG is a proof reading enzyme for mtDNA which was shown in association to Parkinson's disease [88], [89]. POLG knock down mice have increased mtDNA synonymous substitution rates [90].

Heteroplasmy results by microarray at ND4L 10652 were largely not concordant with two separate validation attempts. One MDD subject was shown to have 81% heteroplasmy with microarray, 88% heteroplasmy with allele specific PCR, the C allele with direct sequencing and was not run with SNaPshot. Only two additional subjects were confirmed to have ‘significant’ heteroplasmy levels for this rare variant, 0.47% compared to the microarray resequencing results which were 69% and 58%. These three subjects had psychiatric disorders and were within the M, N and pre HV haplogroups. Further work is required to determine why the microarray T10652C mtDNA variants could not be validated, since the putative carriers had a four-fold higher relative risk of psychiatric disorders compared to controls, and died at younger ages both in control and psychiatric comparisons.

In a study of 18 Alzheimer cases and 19 controls there was a statistically significant difference reported for the T10652 expected reference signal (p<0.05), although it was not specified if it was the C allele substitution [65]. Given the fact that this SNP was not present in 2,704 sequences distributed among major haplogroups and only reported in one L2b individual (Mitokor 568 sequence), the high point prevalence in both our study and the Alzheimer's study is suspect since both studies used Affymetrix mitochondrial resequencing arrays.

With the above caveat in mind, we nevertheless found a common haplotype of four SNPs – T10652C, T14783C, G15043A, and T9540C in 15 of 16 subjects in the M, N and pre HV haplogroups. Individuals with this haplotype cluster separately from the remainder of subjects near the L1 and L2 haplogroups suggesting an ancient origin. It is tempting to speculate that the common haplotype might be a marker for the increased substitutions in brain mtDNA which is associated with younger ages of morbidity in both psychiatric disorder and control groups. The haplotype (T14783C, G15043A, and T9540C) could be further investigated for functional mitochondrial differences. A trend for an association for each SNP was found (T14783C, G15043A, T9540C) suggesting that although these SNPs did not pass permutation corrected p values<0.05, there is a putative haplotype association with risk for psychiatric disorders that can be tested in larger sample sizes.

There was a recent finding that schizophrenia was over-represented in the HV haplogroup with increased relative risk (odds ratio) of 1.8 [64]. Our findings for haplogroup association with psychiatric disorders were significant for pre HV showing increased risk, T showing a protective factor. While based upon a small sample size these findings clearly warrant replication in a larger sample size.

Brain pH showed an association with super haplogroup (U, K, UK) which had a significantly higher pH, i.e. a 1.54 fold decrease in H+ concentration compared to the remaining haplogroups. If haplogroup specific variations in mtDNA can produce alterations of pH levels in human brain, then it could be anticipated that disease associations might also be seen in a larger study. In fact, there was a protective effect of the haplogroup UJKT [91] in Parkinson's disease, which taken together with evidence of higher brain pH in the U, UK, and K subjects might be an indicator of more adaptive brain physiological responses to hypoxia. It has been hypothesized that certain haplogroups are more tightly coupled in the following order from most to least (H>J = T>U>UK) [92]. The haplogroups that are more tightly coupled will generate more reactive oxygen and the less coupled U and UK would have looser coupling efficiency and generate the least ROS in this group. This loose coupling could explain the higher pH, due to less excess mitochondrial oxidation and decreased H+ ion gradients in the outer membrane. A mitochondrial component in diathesis to schizophrenia and mood disorders might involve more tightly coupled OXPHOS processes that occur in H haplogroups on the left of the inequality expression. This tendency towards association of psychiatric disorders with H haplogroups was observed in this study and other studies [58], [64].

Synonymous base pair changes were observed in mtDNA genes in DLPFC: ND2, ND4L, ND6, and Cytb. Three of these ‘associated’ genes ND2, ND4L, and ND6, are located in complex I of the mitochondrion. Associations with complex I mtDNA SNPs such as ND4 T12027C and ND5 C12403T have been reported in schizophrenia [59], [60] and ND1 T3644C mutation in complex I in BD [52]. A decreased expression of complex I mitochondrial transcripts in SZ was recently shown in DLFPC [93]. In addition, a decreased expression of nuclear encoded subunits of complex I was shown in SZ in prefrontal cortex and striatum [94]. This is consistent with inhibition of complex I by various antipsychotics. Haloperidol potently inhibits complex I, followed by chlorpromazine, fluphenazine and risperidone while the atypical neuroleptic, clozapine, did not inhibit complex I activity in mouse brain slices [95]. Neuroleptics such as haloperidol and flupenthixol reduce complex I activity in brain of subjects with SZ [96]. The production of superoxide anion O2−, the principle agent of oxidative stress, is reduced in brain homogenates from rats treated with flupenthixol and reduced in brain tissue and lymphocytes from schizophrenics receiving neuroleptic medication [96]. These studies support the view that a classical neuroleptic such as haloperidol inhibits mitochondrial complex I and reactive oxygen species (ROS) [97]. Complex I deactivation pathophysiology is observed in postmortem brain at both the protein and mRNA levels and the question of whether medications and interactions with mtSNPs requires additional studies. It might be that mtSNPs modulate the responses to antipsychotic treatment at the mitochondrial level as suggested by a recent mtDNA SNP pharmacogenomic study [61].

In summary, this study analyzed mitochondrial DNA variation in brain and found increased mitochondrial DNA substitutions in subjects with SZ compared to controls similar to findings in blood [58] for SZ. Haplogroup associations with risk and protective factors for SZ and BP were found that require further replication. One MDD subject carried essentially a homoplasmic mutation in DLPFC at ND4L T10652C, and two subjects with SZ showed less than 1% heteroplasmy; however, the pathological significance of the low levels of this non synonymous heteroplasmic mutation is unknown. This study replicated that low levels of heteroplasmic non-synonymous mutations are found in the brain [87]. A third finding showed that the UK macro haplogroup maintained a higher pH in response to terminal brain hypoxia. The present findings taken together could be useful in mitochondrial medicine for earlier diagnosis and treatment of brain related disorders based upon recognition of haplogroup and individual susceptibility factors.

## Methods

### Ethics Statement

Informed consent to allow genetic testing was obtained from the next of kin for each sample and the collected samples were approved by the University of California, Irvine Institutional Review Board.

Synonymous and non synonymous substitutions in mtDNA in all coding and control regions were identified in dorsolateral prefrontal cortex (DLPFC) by microarray resequencing of schizophrenia (SZ), bipolar disorder (BD), major depressive disorder (MDD), and control (C) subjects. The demographics for the study are shown for the mitochondrial DNA resequencing analysis (Table 7). A total of 77 subjects (12 with BD, 14 with SZ, 15 with MDD, and 36 controls) were initially studied. The majority of subjects were identified as Caucasian (71), the remaining were African - American (1), Asian (2), Other (2), and Pacific Islander (1). Age, gender, pH, PMI and DNA crossing threshold (Ct) of each subject are summarized in Table 7. The average pH for MDD subjects was slightly higher than all other groups and the mean age for SZ was lower than all other groups. We included subjects with a rapid death, and no known agonal factors related to chronic illness. Rapidity of death was scored according to the Hardy *et al.* scale [98]. A rapid death occurs within 1 hr from agonal onset based upon coroner's office estimates and is rated on the Hardy scale as 1 or 2. For qualitative purposes, examples of agonal factors are medical conditions at the time of death such as coma, pyrexia, skull fracture, hypoxia, dehydration, multi-organ failure, seizures and ingestion of neurotoxic substances [98], [99], [100], [101]. Each case and control was screened by an extensive review of the medical examiner's conclusions, coroner's investigation, medical records, toxicology results, and interviews of the decedent's next-of-kin.

**Table 7:** Demographics of subjects from which DLPFC mtDNA was resequenced.

Group	N	pH		Age (yrs)		Sex	PMI (hr)		DNA (Ct)	
		Mean	SD	Mean	SD	M/F	Mean	SD	Mean	SD
BD	12	6.87	0.16	50	17	9/3	25.1	9.6	29.0	1.1
MDD	15	6.96*	0.19	51	15	11/4	24.6	6.7	30.3	2.2
SZ	14	6.87	0.24	45*	9	11/3	24.0	12.1	30.6	2.5
C	36	6.81	0.19	53	13	31/5	23.0	8.0	30.0	2.2

All subjects had a rapid death and absence of prolonged hypoxia prior to death. An increased pH was found in MDD compared to the C group. Subjects with SZ died at younger age than C subjects. The Ct for extracted DNA was measured by qPCR for genomic DNA and was similar between groups.

Suicidal death occurred in 17 SZ, MDD, and BD cases. Brain samples from SZ, BD, MDD, and controls were collected previously from the Orange County Medical Examiner's Office [102]. Each brain underwent a gross neuropathological examination to rule out lesions, infarcts, and gross abnormalities [103]. The neuropathology written opinion was based upon careful examination of high resolution digital images of 28 coronal slices per whole brain. The 1 cm coronal slabs were frozen at −140°C, placed into sealed bags, and transferred to −80°C storage. A best estimate clinical diagnosis was based upon DSM-IV criteria applied to medical and hospital records, and next of kin interviews conducted by two psychologists, and were reviewed by a senior psychiatrist.

The dorsolateral prefrontal cortex (DLPFC Brodmann areas 9/46) was dissected from coronal slabs on the left side using neuroanatomical landmarks rostral to the corpus callosum from the medial frontal gyrus on a platform cooled with dry ice. The dissected prefrontal regions from each brain were weighed frozen (∼100 mg) and processed. DNA was extracted from brain tissue using a standard Trizol protocol. Briefly, the DNA was extracted from the phenol phase and precipitated with ethanol. The DNA was then washed twice with 0.1 M sodium citrate in 10% ethanol and solubilized in 8 mM NaOH. The extraction technique provided excellent recovery and quality of nucleic acid assessed by qPCR (Table 7).

### Affymetrix Mitochondrial Resequencing Arrays

Genomic DNA obtained from DLPFC was used on Affymetrix GeneChip Mitochondrial Resequencing Arrays 2.0 following manufacturer's protocol (Affymetrix, Santa Clara, CA). Briefly, the mitochondrial resequencing chip has both strands of the entire human mitochondrial coding sequence (15,451 bp) arrayed; both strands of an additional 12,935 bp (84% of coding DNA) are arrayed in duplicate. We used 300 ng of genomic DNA to amplify the mitochondrial genome in three overlapping long PCR fragments and pooled equimolar amounts of amplicon using the protocol from the Affymetrix GeneChip Resequencing Reagent Kit. The reliability of mtDNA resequencing was determined by comparing mtDNA sequence on the same subjects measured twice by the resequencing microarray. The accuracy of the resequencing microarray results was compared to conventional ABI direct sequencing for three subjects.

### Statistical Analysis

Frequencies of assigned allele calls were compared by using the allele chi-square test in PLINK for a case - control analysis of MT chromosome, and running permutation analysis (n = 50,000 permutations) for determining empirical p-values (URL: http://pngu.mgh.harvard.edu/purcell/plink/) [79]. Associations between mtDNA ethnic specific haplogroup variants and postmortem pH values were tested in PLINK.

Tests of substitution rate differences between groups were conducted as follows. The pair wise comparisons of substitutions between SZ, BD, MDD, and controls were calculated with Molecular Evolutionary Genetics Analysis (MEGA software version 4.0) [84] using the Nei-Gojobori method [84], [85], [103]. The probability of rejecting the null hypothesis of strict-neutrality of selection (dN = dS) in favor of the alternative hypothesis (dN<dS) was calculated for the test statistic (dS−dN). The variance of the difference was computed using the bootstrap method (10,000 replicates). Values of p<0.05 were considered significant and tabulated for each group and group frequencies were tested with Pearson's chi-square. An estimation of the transition/transversion bias for each group was also calculated in MEGA4 [84].

Haplogroup assignment and mtDNA transversions/transitions were determined with the MitoMaster database [104], [105] in the Center for Molecular and Mitochondrial Medicine and Genetics. This uses neighbor joining distances to assign haplogroups based upon specific mtDNA haplotype.

Heteroplasmy was screened by microarray calculation with the method of Coon *et al.* [65]. The microarray signal intensity data from all four alleles (A, C, G, and T), on both the sense and antisense strands formed the relative expected allele intensity. The expected allele was called using the revised Cambridge Reference Sequence (rCRS) [106] (Accession # REFSEQ AC_000021.2 gi∶115315570).

### Direct Sequencing and Cloning of ND4L T10652C Variant

To validate the ND4L T10652 variant, PCR was performed on the 17 DLPFC samples that showed the highest levels of heteroplasmy at this locus with the resequencing arrays. These subjects were in the N (N1B1, pre HV) and M (D1) haplogroups. Primers were designed using Primer3 software (Totowa, NJ) (forward 5′-TCGCTCACACCTCATATCCTC-3′ and reverse 5′-GGCCATATGTGTTGGAGATTG-3′). PCR reactions were run on a 2% agarose gel and fragments were cut out and purified using the QIAquick Gel Extraction Kit (Qiagen, Valencia, CA). Sequencing reactions were run using the forward PCR primer and the Big Dye Terminator Kit (Applied Biosystems, Foster City, CA). Products were purified using a DyeEx 2.0 Spin Kit (Qiagen) and analyzed on an ABI Prism 3100 capillary sequencer. Sequences were read in Geneious (Biomatters, Ltd., New Zealand) for alignment around mitochondrial locus 10652. The sample showing the highest heteroplasmy level (81%) by microarray (the MDD sample in haplogroup D1) was validated by capillary sequencing and used to create the T allele clone for the standard curve for SNaPshot analysis. A second sample, showing very low heteroplasmy by microarray, was chosen for the C allele clone for the standard curve. Both clones were used for PCR with the ND4L primers and PCR products were gel purified and cloned into the pCR®2.1-TOPO® vector using the TOPO-TA Cloning® Kit (Invitrogen Life Technologies, Carlsbad, CA). Transformations used One Shot® Mach1™-T1^R^ competent cells and were plated out on LB plates with X-gal and kanamycin. White and light blue colonies were selected and minipreps were purified using the PureLink™ Quick Plasmid Miniprep Kit (Invitrogen) according to the manufacturer's instructions. Clones were tested for correct insert sequence and orientation using the M13 forward and reverse primers.

### SNaPshot Standard Curve

Established clones for the T and C alleles at ND4L 10652 were used to create the standard curve for SNaPshot analysis. The ABI Prism SNaPshot Multiplex Kit (ABI, Foster City, CA) was used according to the manufacturer's protocol with 0.10 pmols of template. Seven dilutions were made for the standard curve: 100% C allele, 83% C allele, 10% C allele, 5% C allele, 1% C allele, .1% C allele and 100% T allele. The SNaPshot single-base extension reactions were run on the 3100 Genetic Analyzer (ABI, Foster City, CA). DNA fragment data was analyzed using the Peak Scanner Software v1.0 (ABI, Foster City, CA) to obtain the ratio of the peak heights corresponding to each allele for the different levels of heteroplasmy in the standard curve. Once a calibration curve was established with varying dilutions of each clone, 24 mtDNA samples from DLPFC that showed varying levels of heteroplasmy with microarray were run using the SNaPshot Multiplex Kit.

### Allele Specific PCR

An allelic specific real-time detection and quantification of mtDNA variants was implemented to more precisely confirm the percentage heteroplasmy observed in the microarray experiment. Low levels of heteroplasmy down to 0.02% were detected using a method derived from Strand *et al.* [107]. This involved an allele specific amplification by PCR [108], followed by the quantification of the amounts of T and C alleles using the Light Cycler 480 (Roche, Indianapolis, IN). The standard curve was constructed from the 10652 clones (see above) using absolute copy number proportions of each allele (500 to 1 million copies). Copy numbers of each allele for the 77 DLPFC samples were then calculated from the standard curve after an allelic specific PCR to determine the unknown “Cp” of each allele. The heteroplasmy was calculated as the % C over the total (C+T). High specificity and sensitivity for the mutant C allele were achieved by blocking non-specific amplification of the wild type allele with an amine blocked T specific primer.
